# 5D imaging via light sheet microscopy reveals cell dynamics during the eye-antenna disc primordium formation in *Drosophila*

**DOI:** 10.1038/srep44945

**Published:** 2017-03-21

**Authors:** Yu Shan Huang, Hui Yu Ku, Yun Chi Tsai, Chin Hao Chang, Sih Hua Pao, Y. Henry Sun, Arthur Chiou

**Affiliations:** 1 Institute of Biophotonics, National Yang-Ming University, Taipei, Taiwan; 2 Department of Life Sciences and Institute of Genome Sciences, National Yang-Ming University, Taipei, Taiwan; 3 Institute of Molecular Biology, Academia Sinica, Taipei, Taiwan; 4 Biophotonics and Molecular Imaging Research Center, National Yang-Ming University, Taipei, Taiwan

## Abstract

5D images of *engrailed (en*) and *eye gone (eyg*) gene expressions during the course of the eye-antenna disc primordium (EADP) formation of *Drosophila* embryos from embryonic stages 13 through 16 were recorded via light sheet microscopy and analyzed to reveal the cell dynamics involved in the development of the EADP. Detailed analysis of the time-lapsed images revealed the process of EADP formation and its invagination trajectory, which involved an inversion of the EADP anterior-posterior axis relative to the body. Furthermore, analysis of the *en-*expression pattern in the EADP provided strong evidence that the EADP is derived from one of the *en*-expressing head segments.

The larval eye-antenna disc (EAD) in *Drosophila* has served as a popular experimental system for the study of many biological processes^1^,^2^,^3^,^4^,^5^,^6^. It develops into the adult compound eyes, ocelli, antenna, maxillary palp, and the head capsule that surrounds these organs^7^. When the larva is first hatched, the EAD can be morphologically recognized to contain about 70 cells^8^. Several studies have mapped and followed the development of the precursors for the EAD (eye-antenna disc primordium, EADP) during embryo development^9^,^10^,^11^,^12^,^13^,^14^.

The development of the EADP is related to the morphogenesis of the *Drosophila* visual system, which is composed of the larva eye (the Bolwig’s organ), the adult eye (developed from the EADP) and the optic lobe of the brain. The *Drosophila* visual system is thought to be originated from the dorsal head region in the blastoderm embryo. Analysis using gynandromorphs and somatic clones^15^,^16^ estimated the number of founder cells for each EAD in blastoderm stage to be 23 and 6, respectively. Fate mapping experiments, by gynandromorph mapping, photoactivation tracing, and cell ablation, suggest that the EADP can be tracked back to the dorsal lateral head region in blastoderm embryo^7^,^10^,^13^,^17^. In stage 4, *sine oculis (so*) is expressed in a broad unpaired head area, known as the visual primordium, that is believed to give rise to the *Drosophila* visual system^11^,^14^,^18^,^19^. In stage 5, it partitions to form bilaterally symmetrical procephalic lobes^14^. In stage 11, the optic lobe placode is formed from the lateral part of the visual primordium^11^. In stages 12 and 13, the optic lobe placode invaginates by apical constriction and then splits into the optic lobe (defined by *so* expression) and Bolwig’s organ (defined by mAb22C10 and FasII expression)^11^. The EADP can be identified by the expression of the Pax6 gene *eyeless (ey*) in stage 12 as two symmetrical regions on the dorsolateral surface in the head^14^,^19^. Ey expression in the EADP continues throughout the embryonic stage^19^. The Pax gene *eye gone (eyg*) and its sister gene *twin of eyegone (toe*) are expressed in the EADP beginning in stage 15^20^,^21^. Lineage mapping showed that *ey* and *eyg* expressing cells contributed to the entire EAD^22^. Hence, the EADP can be identified by *ey, eyg* and *toe* expressions.

Although the EADP is thought to be derived from the visual primordium, which is defined by *so* expression, *ey* and *so* expressions do not overlap^23^. Consistently, loss of *so* does not affect *ey* and *toy* expressions and the formation of the EADP^24^. Other genes in the retinal determination network, namely, *dachshund (dac*) and *eyes absent (eya*), are not expressed in the EADP^23^. These observations strongly suggest that the EADP formation is independent of the retinal determination network genes *so, dac* and *eya*. Its origin and the course of formation need further clarification.

Earlier studies of the morphological change of head primordium in late embryonic period suggested that the EADP invaginates in stage 15 to form a V-shape structure^11^,^12^,^19^,^23^,^25^, later fused with other head segments (e.g. the antennal segment)^12^,^13^, and subsequently evaginated from the posterior dorsal pouch as a sac-like structure before hatching^11^,^12^. However, these earlier studies of the EADP were hindered by the lack of specific marker and relied on histology or the general imaginal cell marker *escargot (esg*)^13^,^26^,^27^. Furthermore, the organization and movement of the EADP were deduced from snapshots of individual embryos at discrete time points, rather than from continuous observation of a single embryo. The movement of EADP precursors is particularly difficult to follow because the embryonic head region undergoes rapid and complicated morphogenetic movements (known as head involution) in stage 15.

In this study, we examined fluorescence reporters to mark and track EADP cells via light sheet microscopy^28^,^29^, which provided the desired 5D (three dimensional spatial, in conjunction with temporal and multi-fluorescence channel) images. The extraordinary capability to achieve large field of view, fast acquisition speed and great penetration depth of the dual-illumination light sheet microscopy enabled us to capture and analyze the cellular dynamics of the EADP formation and movement.

## Results

### Markers for the EADP

**Figure 1:**
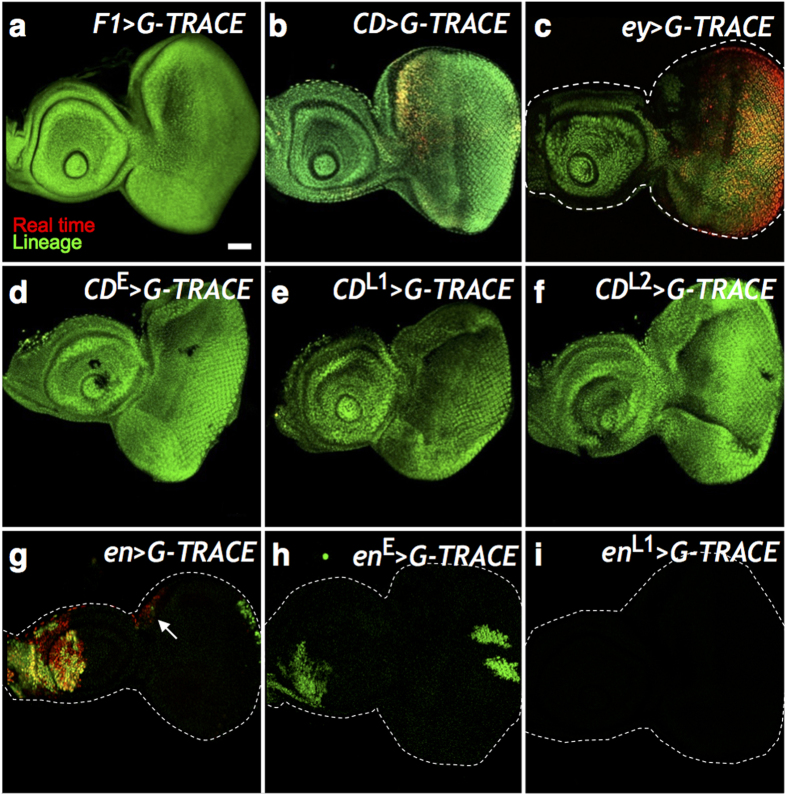
Stage-specific lineage analysis. The G-TRACE reporters RFP and GFP were driven by (**a**) *F1-GAL4 (F1* > *G-TRACE*), (**b**) *CD-GAL4 (CD* > *G-TRACE*), and (**c**) *ey-GAL4 (ey* > *G-TRACE*). Late third instar (L3) EAD were examined. The RFP (red) represents real time expression, while GFP (green) represents lineage expression. (**a**) *F1* was expressed only in embryo, therefore showed no real time expression in the L3 EAD. (**b**) *CD* real time expression mimics *eyg* expression in the equator and the anterior dorsal region in the L3 eye disc. (**c**) *ey* real time expression in the L3 eye disc. (**a–c**) The lineage expression (green) of *F1, CD*, and *ey* comprised entire EAD. (**d–f**) Stage-specific *CD* > *G-TRACE* in embryonic, L1 and L2 stages (designated as *CD*^E^, *CD*^L1^ and *CD*^L2^, respectively). *CD*^+^ cells in the embryonic, L1, and L2 stages contributed to the entire EAD. Since the GAL80^ts^ blocked the GAL4 activity in L3, there was no real time expression in the L3 EAD. (**g**) The *G-TRACE* reporters driven by *en (en* > *G-TRACE*). *en*^+^ cells contributed to the posterior compartment of the antenna and eye disc. Real-time *en* expression in L3 was in the posterior compartment of the antenna disc and in the ocellar region (white arrow) in the eye disc. (**h–i**) Stage-specific *en* > *G-TRACE* in embryonic and L1 stages (designated as *en*^E^ and *en*^L1^, respectively). *en*^+^ cells in the embryonic stage contributed to the posterior compartment of the antenna and eye discs. Note the inverted anterior-posterior axis of antenna disc relative to that in the eye disc. *en*^+^ cells in L1 did not contribute to the L3 EAD, suggesting that *en* was not expressed in L1 EAD. Scale bar is 50 μm and the anterior is to the left in all frames.

The enhancers in the *eyg-toe* locus have been dissected^22^, of which the *CD* enhancer mimics the *eyg* expression from embryonic stage to the larval EAD, the *F1* enhancer is expressed in the EADP, but not in later stages, and the *EX* enhancer is expressed in the EADP and first instar (L1) eye disc. Lineage tracing^30^ via *CD* enhancer to drive the induction of clones has shown that the *CD*-expressing (*CD*^+^) cells contributed to the entire EAD^22^. We used the G-TRACE method^31^ to confirm the lineage distribution. G-TRACE showed that *F1, CD*, and *ey* lineage cells contributed to the entire EAD (Fig. 1a–c). Our results showed that *eyg* expression (represented by the *F1* and *CD* enhancers) and *ey* expression in the embryonic stage can be used to identify the EADP. We further combined *GAL80*^*ts*^ 32 and used temperature shifts to induce clones in different developmental stages. The results showed that the lineage of *CD*^+^ cells in the embryonic, L1, and second instar (L2) stages contributed to the entire EAD (Fig. 1d–f), indicating that *CD* is expressed in all cells in the EADP and continued to be expressed throughout the EAD development. The *CD-GAL4* induced lineage clones in embryonic stage contributed to the entire L3 EAD, including the disc proper and the peripodial membrane (Supplementary Fig. S1). The result indicated that the *CD*-expression in the EADP in the embryonic stage included all the EAD progenitor cells. Therefore, we used the *CD* enhancer as a marker for the EADP.

However, the *CD* enhancer is not activated until late stage 14; hence, it cannot be used to trace the earlier stages of the EADP development. To trace the earlier stages of the EADP development, we used *en-GAL4* driven *UAS-H2B-RFP (en* > *RFP*). In contrast to previous report^12^, *en* is expressed in the EADP. G-TRACE showed that *en*-expressing (*en*^+^) cells contributed to the posterior compartment of the antenna disc, ocellar region, and the posterior part of the eye disc (Fig. 1g). The temperature shift experiments of *en-GAl4* driven *G-TRACE (en* > *G-TRACE*) showed that cells expressing *en* in the embryonic stage *(en*^E^) contributed to regions same as the *en* > *G-TRACE* result (Fig. 1h). Interestingly, *en*^+^ cells in L1 (*en*^L1^) did not contribute to the EAD at all (Fig. 1h), suggesting that the *en* expression in the EAD is interrupted during L1 and resumed in later larval stages (Fig. 1h,i).

Since *en* expression is not limited to the EADP^12^,^33^,^34^,^35^,^36^,^37^, we used live-imaging of *Drosophila* embryos to simultaneously follow the movement of both *CD*^+^ and *en*^+^ cells. When the *CD*-*GFP* expression appeared in the EADP, we traced backward in time to define the *en*^+^ cells that were associated with the EADP. These cells are termed the EADP-engaged *en*-expressing cells (EECs). Since *en* expression (from late stage 8)^33^ in the procephalic region precedes *ey* (from stage 12), EECs potentially can allow us to trace the earliest stage of the EADP formation. However, the *en* > *RFP* expression in the head region was so weak in the early stage, that we were able to trace only as early as stage 13.

### The origin and movement of EECs through a 5-phase process

**Figure 2:**
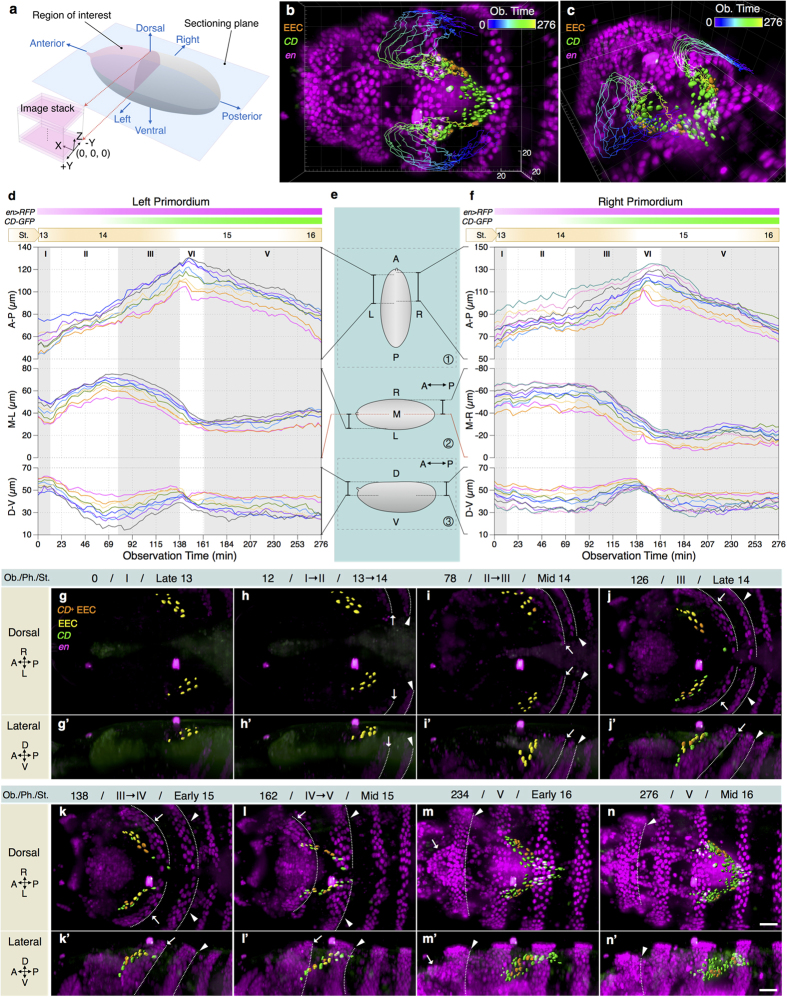
The origin and movement of EECs. (**a**) A schematic illustration of the region of interest, embryo orientation and the 3D coordinates of an image stack. The imaging plane was parallel to the sectioning plane. (**b**) The trajectories of EECs, over the observation time 0 to 276 min, displayed with the 3D fluorescence patterns of *en* > *RFP* (magenta) and *CD-GFP* (green) in dorsal view. EECs are labeled in orange. (**c**) A tilted view of the figure in (**b**). (**d**) A composite chart of the trajectories of EECs in the left primordium, decomposed along 3 mutually orthogonal axes, namely, the anterior-posterior (A-P), the medial-lateral (M-L), and the dorsal-ventral (D-V) axes. The timing of *en* > *RFP* and *CD-GFP* expression and the corresponding developmental stages are marked on top. (**e**) The schematic diagrams showing the locations, the orientations, and the ranges associated with the corresponding diagrams in (**d**) and (f). Diagrams ① and ② represent an embryo in the dorsal view while diagram ③ shows the corresponding lateral view. (**f**) A similar chart as (**d**) for the right primordium. (**g**–**n**) The dorsal views of EADP cells movement from embryonic stage 13 to stage 16. The observation time points (Ob. Time, listed on the top of the corresponding figures) of the images from (**g–n**) are 0, 12, 78, 126, 138, 162, 234, and 276 min, respectively. (**g’**–**n’**) The lateral view of the left primordium corresponding to figures (**g–n**). The corresponding phase (Ph.) and the equivalent embryonic stage (St.) are listed next to the observation time. The dorsal ridge and the first thoracic segment are indicated by “ → ” and “▸”, respectively. Their posterior boundaries are indicated by dotted lines. In frames (**m,m’**), (**n**,**n’**), the dorsal ridge has involuted inside the embryo so that only the posterior boundaries of the first thoracic segment are visible. Grid size in the background is 20 μm in (**b,c**). Scale bars are 10 μm in frames (**n**) and (**n’**). Frames (**g–n**) and (**g’–n’**) are in the same scale.

We followed simultaneously the *CD*^+^ and *en*^+^ cells via dual-illumination light sheet microscopy (Supplementary Fig. S2) and quantitatively analyzed the 5D image data to study the cell dynamics of EADP cells. Our region of interest was the dorsal half of the head region, as shown in Fig. 2a. Each dual fluorescence channel 3D image was acquired within 40 seconds, every 3 minutes, for a total period of 4 to 7 hours, depending on the time point when the embryo started to wriggle within the vitelline membrane to disable clear imaging (Supplementary Movie 1 and Supplementary Fig. S3). EECs were tracked back to their initial locations. The 3D trajectory and the surface rendering of each EEC and *CD*^+^ cell were reconstructed from their first appearance through the last trackable observation time point (Fig. 2b and c). Although the numbers of EECs in the left and the right EADP in a single embryo were not identical (4 to14 cells in each primordium in the 6 embryos that we have analyzed), their migration patterns in different embryos were consistent (Supplementary Fig. S4).

Based on our observations, we propose that the course of the EADP formation can be phenomenologically divided into the following 5 phases (Fig. 2d,f; Supplementary Movie 2).

Phase I (from observation time 0 to 12 min.): EECs emerged as two *en*^*+*^ stripes, one on each side near the periphery of the procephalic regions (Fig. 2g) and showed slight random movements. The dorsal ridge and the epidermal segments were barely discernible from the dorsal view due to their weak fluorescence signals and their relatively ventral position.

Phase II (from observation time 13 to 78 min.): In this phase, dorsal closure and head involution began, and a few EECs started to express *CD*-*GFP*. The dorsal ridge and *en*^+^ epidermal segments appeared (Fig. 2h). EECs started to migrate anteriorly and slightly ventrally, as well as toward the midline (Fig. 2g,g’ and also d and f).

Phase III (observation time 79 to 135 min.): The dorsal ridge and the epidermal segments began to fuse at the dorsal midline and moved anteriorly. EECs continued to move toward the dorsal midline (Fig. 2i,i’). More EECs expressed *CD*-*GFP*.

Phase IV (observation time 136 to 162 min.): The dorsal ridge and the epidermal segments moved anteriorly and over the cephalic region (Fig. 2k,k’). EECs rearranged into slender stripes along the A-P axis, and reversed their anterior trajectories to move posteriorly. EECs also moved ventrally, as if they were pressed downward by the sliding of the dorsal ridge above them. At the end of this phase, EECs were completely internalized (Fig. 1l,l’). A few non-EEC *CD*^+^ s appeared around EECs (Fig. 2l,l’) and migrated collectively with EECs (Supplementary Movie 3).

Phase V (observation time 163 to 276 min.): EECs and the *CD*^+^ cells kept moving posteriorly after being overwhelmed by the epidermal segments. The epidermal sheets fused completely at dorsal midline as the dorsal closure terminated at the end of stage 15. All the EECs expressed *CD*-*GFP* at the observation time 234 (Fig. 2m,m’). The number of *CD*^+^ cells increased significantly (Supplementary Fig. S5a), and the sac-like structure of the EADP was readily distinguishable (Fig. 2n,n’).

During the observation period, the fluorescence intensity of *en* > *RFP* in EECs increased with time before internalization, but slightly decreased thereafter (Supplementary Fig. S5b). In contrast, the *CD-GFP* intensity in EECs continuously increased with time. Severe fluctuations of fluorescence signals were observed during the head involution because of the rapid morphogenetic movement. It is noteworthy that we did not see any cell division in the EADP throughout the period of observation, indicating that the increase in *CD*^+^ cells is not due to cell division but due to the expression of *CD* (representing *eyg* transcription). No mitotic cells (stained by anti-phospho-histone H3; pHH3) were detected to co-localize with *CD*^+^ cells (Supplementary Fig. S6). No spatial preference of the position of new non-EEC *CD*^+^ cells relative to EECs was discerned. The spatial distribution of the subsequently-formed non-EEC *CD*^+^ cells do not seem to bear any correlation relative to that of EECs.

### Spatiotemporal mapping of *CD* expression during the course of the EADP formation

**Figure 3:**
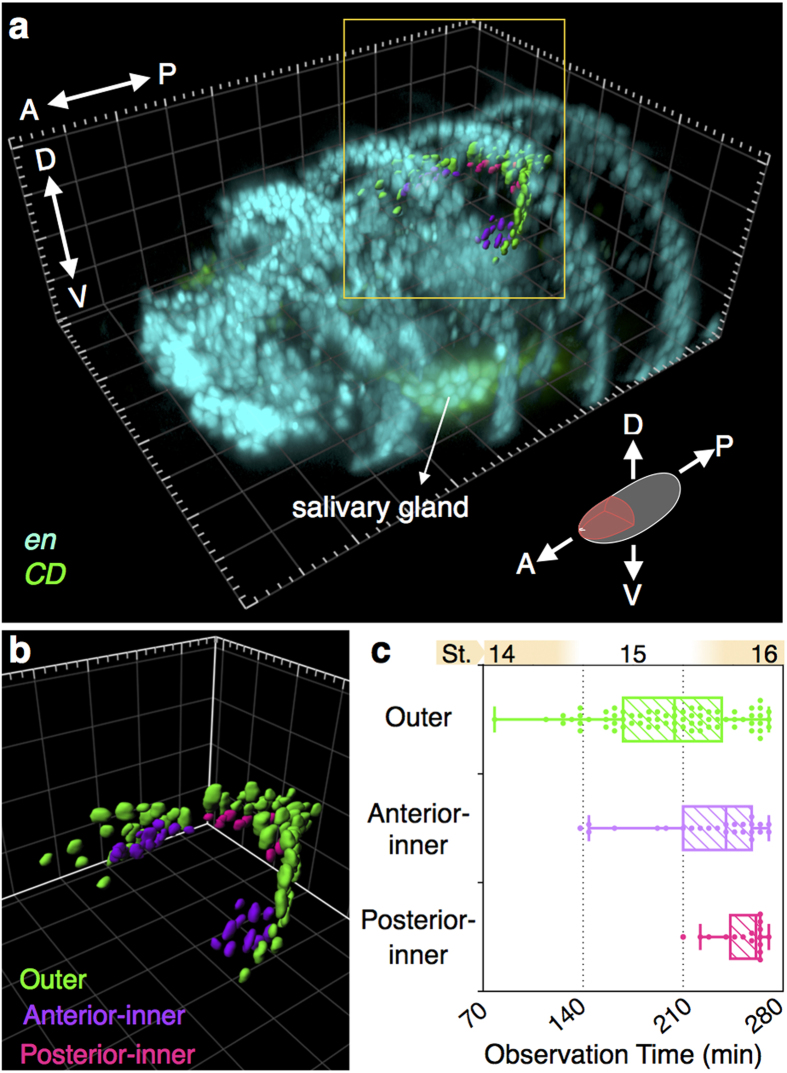
The spatiotemporal relationship of *CD* expression in the EADP. (**a**) A 3D volume rendering of an *en > RFP* (cyan) and *CD-GFP* (green) co-expressing embryo from a tilted view at stage 16. The orientation and the imaging area of the data is displayed as the pink region in the schematic embryo. *CD*^+^ EADP cells are shown in 3D surface rendering and categorized into outer, anterior-inner, and posterior-inner groups, which are color-coded in green, purple, and pink, respectively. Some ventral *en*^*+*^ *CD*^*+*^ cells belonged to the salivary gland. (**b**) An enlarged version of the EADP without showing the *en-* and *CD*-expression patterns. (**c**) The distribution of the onset of *CD* expression of EADP cells. The data points outside the whiskers are regarded as outliers. Grid size in the background in (a) and (b) is 20 μm. Abbreviation: A, anterior; D, dorsal; St., embryonic stage.

**Figure 4:**
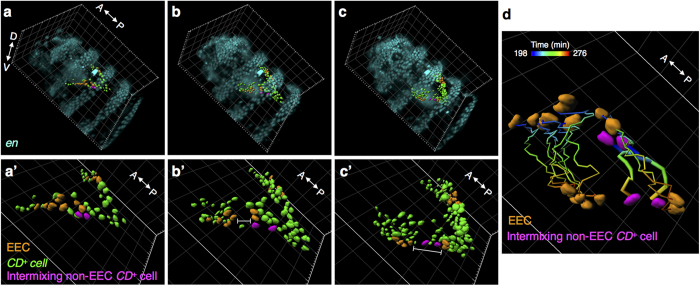
The cell intermixing in EECs during the EADP formation. (**a**–**c**) The 3D volume rendering of fluorescence signals from an *en > RFP CD-GFP* co-expressing embryo at observation time 198, 240, and 276 min in tilted view. The corresponding embryonic stage was about stage 16. To highlight the EADP, *CD*^+^ EADP cells are shown in surface rendering. The dataset is the same as in Fig. 2. (**a’–c’**) Enlargements of *CD*^+^ EADP cells in (**a–c**), respectively; *CD*^+^ EECs are shown in orange, and *CD*^+^ cells are shown in green. Two intermixing non-EEC *CD*^+^ cells are highlighted in magenta in the left primordium. Gaps between EECs in the left primordium are indicated by the white I-bars in (**b’**) and (**c’**). (**d**) The trajectories of EECs and two intermixing cells from time points 198 to 276 min. The temporal sequence of each trajectory is color-coded. The surface renderings of EECs and the intermixing non-EEC *CD*^+^ cells, are labeled in orange and magenta, respectively, at both the starting point and the end point. The trajectories of intermixing non-EEC *CD*^+^ cells are highlighted in thicker lines. Grid size in the background of all frames is 20 μm.

The number of *CD*^+^ cells increased progressively without any cell division, suggesting that the cells progressively expressed *CD*. We examined the spatiotemporal pattern of *CD* expression during the course of the EADP formation, and analyzed the correlation between the expression timing and spatial distribution within the EADP. In stage 16, the EADP cells can be characterized as belonging to either an outer, an anterior-inner, or a posterior-inner group based on their locations within the the EADP structure (Fig. 3a). From the EADP in stage 16, we back-traced the origin of each *CD*^+^ cells in each group and analyzed their onset of *CD* expression time (Fig. 3b and Supplementary Movie 4). The result shows that most of the cells in the outer layer expressed *CD-GFP* earlier than those in the inner layer, and cells in the posterior-inner group expressed *CD-GFP* much later than those in other groups. The relationship between the onset of *CD-GFP* expression and the spatial distribution of *CD*^+^ cells in the EADP was compatible in the three embryos we analyzed (Supplementary Fig. S7). Most, but not all, of the EECs were confined in the outer layer (Fig. 4). Whether the temporal expression of *CD* determined its final spatial destination, or its location determined the timing of expression, awaits further study.

### EECs allow cell intermixing during the EADP formation

Before the internalization, EECs generally moved collectively as a cluster. However, we observed that in some cases a group of EECs could be trespassed by non-EEC *CD*^+^ cells during the EADP morphogenesis (Fig. 4a–c). The trajectory of each cell, examined from a tilted angle, revealed how the non-EEC *CD*^+^ cells trespassed the continuity of EECs (Fig. 4d and Supplementary Movie 5). Unfortunately, the severe image blurring, due to the embryonic wriggling at later times, disabled further analysis. Surface rendering, at those time points, of EADP images which were not affected seriously by sample wriggling were selected and reconstructed (Supplementary Fig. S8). The morphological change of the EADP indicates that a folding process had taken place to condense the structure of the EADP.

## Discussion

We monitored the course of the EADP formation in *Drosophila* embryos from stage 13 to stage 16 through long-term 5D live imaging, which provided unprecedented clear observation of the cell dynamics during the EADP formation. Our lineage analysis showed that the *CD* enhancer from the *eyg* gene can be used as a faithful marker for the EADP. Although *CD* expression begins only in stage 15, the use of *en* > *RFP* allowed us to trace EECs, hence the formation of the EADP, to as early as stage 13. The position and the migration path of EADP cells were consistent with the expression of *ey*^14^,^19^. Previous studies^23^,^38^,^39^ indicate that various genes, including the *eyeless (ey*), twin of eyeless (*toy*), *sine oculis (so*), *eye gone (eyg*), *homothorax (hth*) and *teashirt (tsh*) are expressed uniformly in the EADP in the first instar larval stage (L1). Gene expressions are not restricted in the eye and the antenna until the second instar larval stage (L2). The EADP has been suggested to be a homogeneous structure in L1, which later segregate to form the eye and the antennal primordium from L2. Thus, specific markers for the eye and the antenna are not available in the embryonic stage.

**Figure 5:**
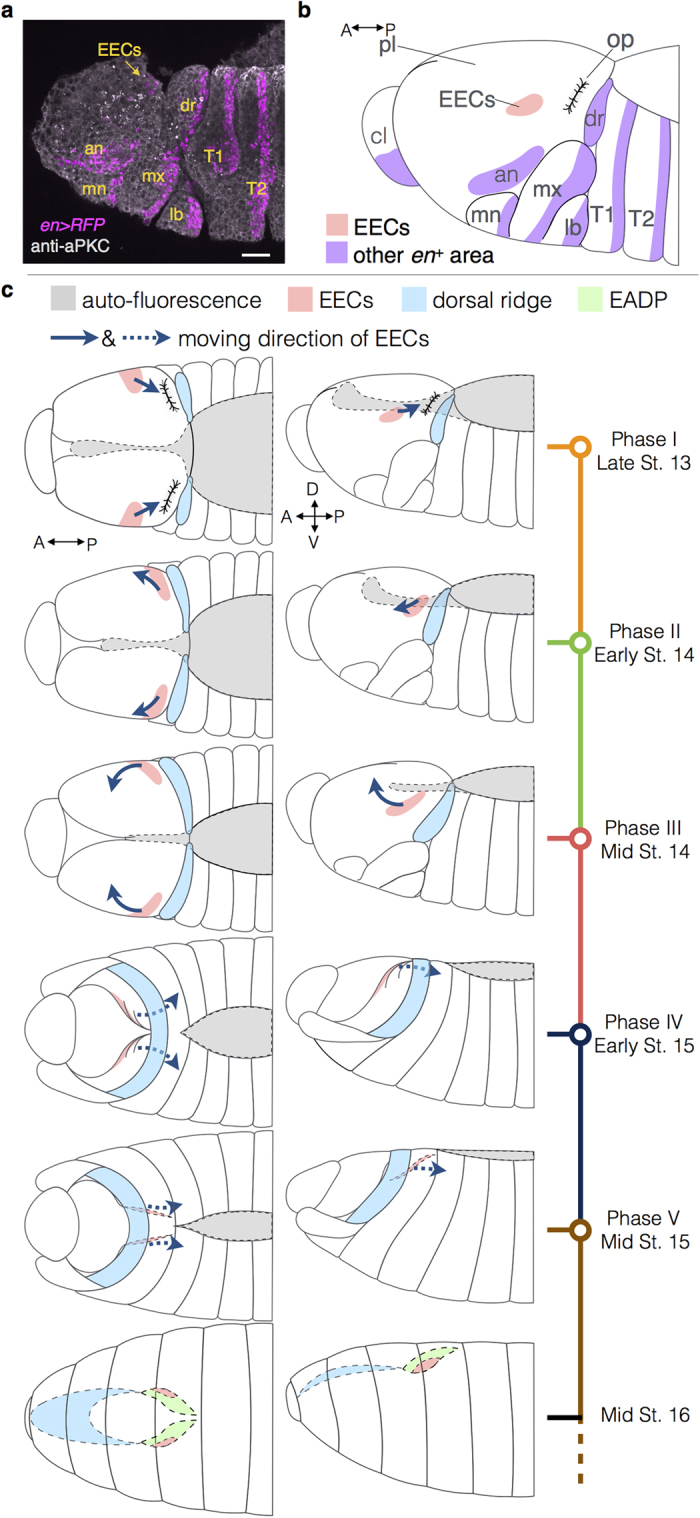
A schematic summary of the location and migration pattern of EECs. **(a)** The *en* expression pattern in lateral view in late stage 13. Scale bar: 20 μm. (**b**) A schematic diagram of the *en* expression pattern in (**a**). EECs and several landmarks, including other *en*^+^ sections in procephalic ectoderm (highlighted in purple) and the invagination of optic lobe, are indicated. EECs localize anteriorly adjacent to the optic lobe without connections to other *en*^+^ areas. (**c**) Migration patterns of EECs and the dorsal ridge in different phases in dorsal (left column) and lateral (right column) views. The corresponding embryonic stages are listed below the phase in timeline labels. Solid arrowed lines indicate the direction of EECs’ movement along the surface of the procephalic lobe, whereas the dashed arrowed lines indicate the EECs’ movement in the interior of the embryo, as well as their subsequent interior movement to form part of the EADP (highlighted in green and marked with dashed borders). The areas shaded in gray represent the auto-fluorescence in the amnioserosa and serve as a reference of the embryo morphogenesis in different stages. Anterior is to the left in all figures. Abbreviation: St., embryonic stage, pl, procephalic lobe; op, optic lobe; dr, dorsal ridge; an, antennal; cl, clypeolabral; mn, mandibular; mx, maxillary; lb, labial; T1, the first thoracic segment; T2, the second thoracic segment.

The origin of EECs in stage 13 is spatially separated from the *en* antenna stripe and anteriorly adjacent to the optic lobes (Fig. 5a, Supplementary Fig. S9, and Supplementary Movie 6), which is consistent with the area of the presumptive eye field described in previous studies^19^,^40^,^41^. Although the EEC and the *en* antennal stripe arise at different locations at the first sign of *en > RFP* that we can detect, we cannot fully rule out the possibility that the two groups of cells share a common origin before *en > RFP* becomes detectable.

The migration pattern of EECs shows that the invagination of the EADP does not follow the invagination of optic lobes in stage 14 as described previously^40^, and neither does it through evagination from the dorsal pouch via involution of the dorsal ridge as previously proposed^12^. Rather, EADP cells move into the interior of the embryo with the aid of the relative motion between the procephalic lobe and the dorsal ridge during the head involution, such that the EADP is pushed under the dorsal ridge. (Fig. 5b).

EECs form a part of the EADP. They were located in a posterior lateral position in the EADP in stage 14 and gradually changed to an anterior lateral position in the EADP. The spatial arrangement of EECs changed from a transverse orientation (approximately parallel to the L-R axis) to a longitudinal orientation (approximately parallel to the A-P axis) during phase I through mid-phase IV before the internalization. We propose a transposition model (Supplementary Fig. S10) to explain why the anterior-posterior orientation of the antenna disc is reversed relative to the body.

After the EADP internalization, we observed in some (but not all) cases that EECs got split into several cell groups due to insertion of non-EEC *CD*^+^ cells as they moved posteriorly in stage 15 and 16. The allowance of separation in EECs conflicts with the absence of cell intermixing between compartments reported earlier^42^,^43^. The phenomenon suggests that the boundary property of the *en*-defined posterior compartment may not be strictly retained during the EADP morphogenesis.

Whether the EAD has a single-segmental or multi-segmental origin has been a long unsettled issue^7^,^12^,^34^,^35^,^44^. Since *en* is expressed in the posterior compartment of all embryonic segments, it has been used in the analysis of the segmental origin of the embryonic head region^12^,^35^,^44^. Our G-TRACE results showed that the *en*^*+*^ cells in the embryonic stage contributed to the posterior compartment of the antenna disc and the posterior part of the eye disc (Fig. 1). Relevant to the eye and antenna, *en* has expression in a pair of antennal stripes and a pair of antennal spots^12^,^34^,^35^; the former was regarded as contributing to the antennae, and the latter was regarded as derived from the *en* antennal stripes in stage 13. We have not observed the *en* antennal stripes fuse with the EADP, as defined by *CD-GFP* expression. EECs are the only *en*^+^ cell group in the EADP. In newly hatched first instar larva, the eye and antennal portion of the EAD has 42 and 35 cells, respectively^8^. In our observation, the number of *CD*^+^ cells reached a plateau of 85–90 per primordium in stage 16, while the number of EECs within the primordium remained at about 10 per primordium since stage 15. These observations show that there is no further contribution from non-EEC *en*^+^ cells to the formation of the EADP after stage 16. However, non-EEC *CD*^+^ cells before late stage 14 were not trackable, so the possibility that non-EEC *CD*^+^ cells originated from multi-head segments in an early time cannot be fully excluded. Overall, these results strongly suggest that the EADP is derived from one of the *en*^+^ head segments, which is defined by EECs.

## Methods

### Fly Strains

*en2.4-GAL4*^45^*, UAS-G-TRACE*^31^, and *tubP-GAL80*^*ts*^ 32 were from the Bloomington stock center. *CD-GFP, CD-GAL4*, and *F1-GAL4* have been reported^22^. *ey-GAL4*^46^ was a gift from Walter Gehring (University of Basel, Switzerland). *ubi-GFP-CAAX*^47^ was from the KYOTO Stock Center (DGRC). All fly grown and crosses were performed according to the standard procedure at 25 °C unless otherwise noted.

### Experimental procedure for stage-specific G-TRACE analysis via temperature shift

For stage-specific lineage tracing, *CD-GAL4*; *tub-GAL80*^*ts*^ or *en-GAL4*; *tub-GAL80*^*ts*^ virgins were crossed with *UAS-G-TRACE* males. Flies were allowed to lay eggs for 4 hours in 17  °C or 29 °C in all stage-specific experiments. The lineage distributions in EAD were examined in L3. A diagram to illustrate experimental principles and timelines is shown in Supplementary Fig. S11. Fixation and staining of larval EAD were performed as described previously^48^. Fixed EAD were imaged by Zeiss LSM780. The morphology of each EAD was determined by differential interference contrast (DIC) image.

### Experimental procedure for 5D imaging via dual-illumination light sheet microscopy

To track the *en > RFP CD-GFP* co-expressing cells, *en-Gal4* and *CD-GFP*; *UAS-H2B-RFP* were crossed to generate the embryos co-expressing *en > RFP* and *CD-GFP*. The embryos were dechorinated at 7 h after egg deposition (AED), which was about embryonic stage 12. The dechorinated embryos were gently mixed with 0.5% agarose with low gelling point agarose (Sigma Aldrich, A9045) at 30˚C. The sample mixture was molded by a capillary with ~1 mm diameter and pushed out after congealing. The mounted embryo was immersed in a PBS-filled chamber and imaged by dual-illumination light sheet microscope at 9 h AED from dorsal view. A 3D image stack was acquired (with an image acquisition time of 40 sec.) every 3 min. The observation and image recording were stopped when the embryos started to wriggle. All imaging experiments were performed at 25 °C.

### Image processing and quantification

5D images were processed and presented via Imaris (Bitplane Inc.). EADP cells were identified by the *CD*-expressing V-shaped structure in the dorsal embryo in stage 16. *en > RFP CD-GFP* co-expressing cells were distinguished by fluorescence co-localization. Some *en > RFP CD-GFP* co-expressing cells belonging to the salivary glands and the foregut were not analyzed. EADP cells were manually tracked back to the initial observation time from the image stacks to quantify the *CD*-expression time and the *CD*^+^ cell number in each trackable time point. The inner and the outer layers of EADP cells were classified by their positions in stage 16 when the two-layer structure became obvious. The anterior and posterior sections of the inner layer were demarcated by the waist of the EADP (Supplementary Fig. S7).

## Additional Information

**How to cite this article**: Huang, Y. S. *et al*. 5D imaging via light sheet microscopy reveals cell dynamics during the eye-antenna disc primordium formation in *Drosophila. Sci. Rep.* **7**, 44945; doi: 10.1038/srep44945 (2017).

**Publisher's note:** Springer Nature remains neutral with regard to jurisdictional claims in published maps and institutional affiliations.

## Supplementary Material

Supplementary Information

Supplementary Video 1

Supplementary Video 2

Supplementary Video 3

Supplementary Video 4

Supplementary Video 5

Supplementary Video 6
