# Supplementary material for: 5D imaging via light sheet microscopy reveals cell dynamics during the eye-antenna disc primordium formation in Drosophila
Source: Sci Rep. 2017 Mar 21;7:44945. doi: 10.1038/srep44945 (PMC5359570; doi:10.1038/srep44945)
Supplement: Supplementary Information [file srep44945-s1.pdf]

5D imaging via light sheet microscopy reveals cell dynamics during the eye-antenna disc primordium formation in *Drosophila*.

Yu Shan Huang[1]**, Hui Yu Ku**[2, 3]**, Yun Chi Tsai**[1]**, Chin Hao Chang**[1]**, Sih Hua Pao**[1]**, Y. Henry Sun**[2, 3 ,*] **and Arthur Chiou**[1, 4, **]

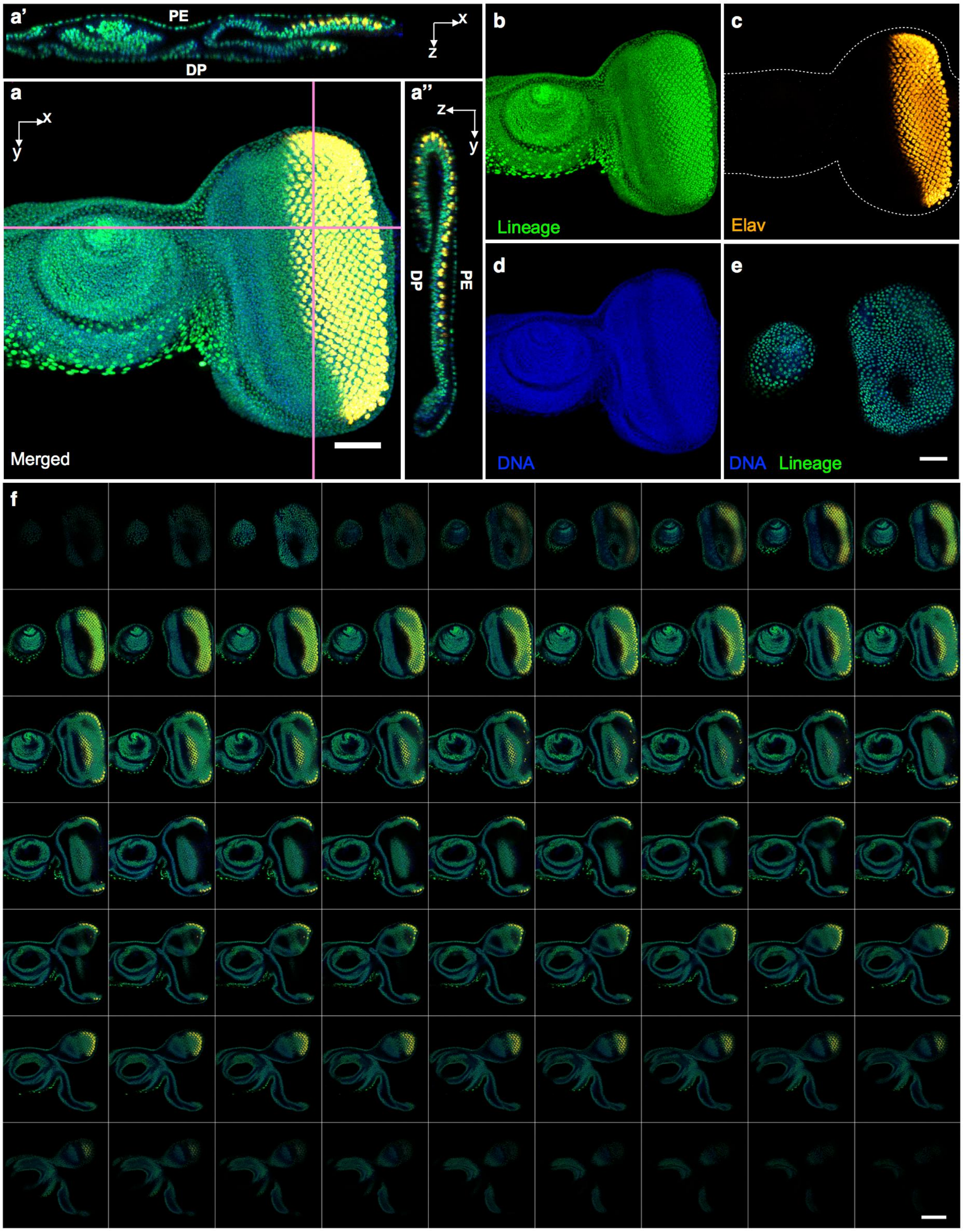

Figure S1. The distribution of CD^{+} lineage cells in a L3 EAD. (**a**) The distribution of CD^{+} lineage clones induced in embryonic stage. The stage-specific *CD>G-TRACE* was carried out following the experimental procedure described in the figure captions of Fig. 1 and Supplementary Fig. S11. (**a'**) and (**a''**) show the orthogonal views (xz and yz planes) of the image shown in (a), sectioned along the pink lines in (a). (**b**), (**c**), and (**d**) The expression patterns shown in green, orange, and blue represent the lineage GFP-reporter, anti-Elav (Developmental Studies Hybridoma Bank, Rat-Elav-7E8A10, 1:500), and DNA staining (DAPI, Sigma-Aldrich D9564, 0.5 μg/ml), respectively. The real-time RFP reporter of *CD>G-TRACE* was not expressed; and hence, was not displayed. These results clearly indicate that CD^{+} lineage clones induced in embryonic stage contributed to the entire L3 EAD. Abbreviations: PE, peripodial epithelium; DP, disc proper. (**e**)

The maximum intensity projection (MIP) of the DNA staining and embryonic stage-specific *CD>G-TRACE* in peripodial epithelium. To isolate the fluorescence signals of the peripodial epithelium from the disc proper, only 7 out of 69 cross-section images containing most of the peripodial cells were composited. The image demonstrated that *CD-GAL4* induced lineage clones in embryonic stage contributed to most of the peripodial cells. (**f**) A montage image of 2D optical-sectioning slices at different depths, separated by 1 μm, to show that *CD*-lineage clones covered the entire disc proper and peripodial epithelium. Scale bars are 50 μm in (a) and (e), and 100 μm in (f).

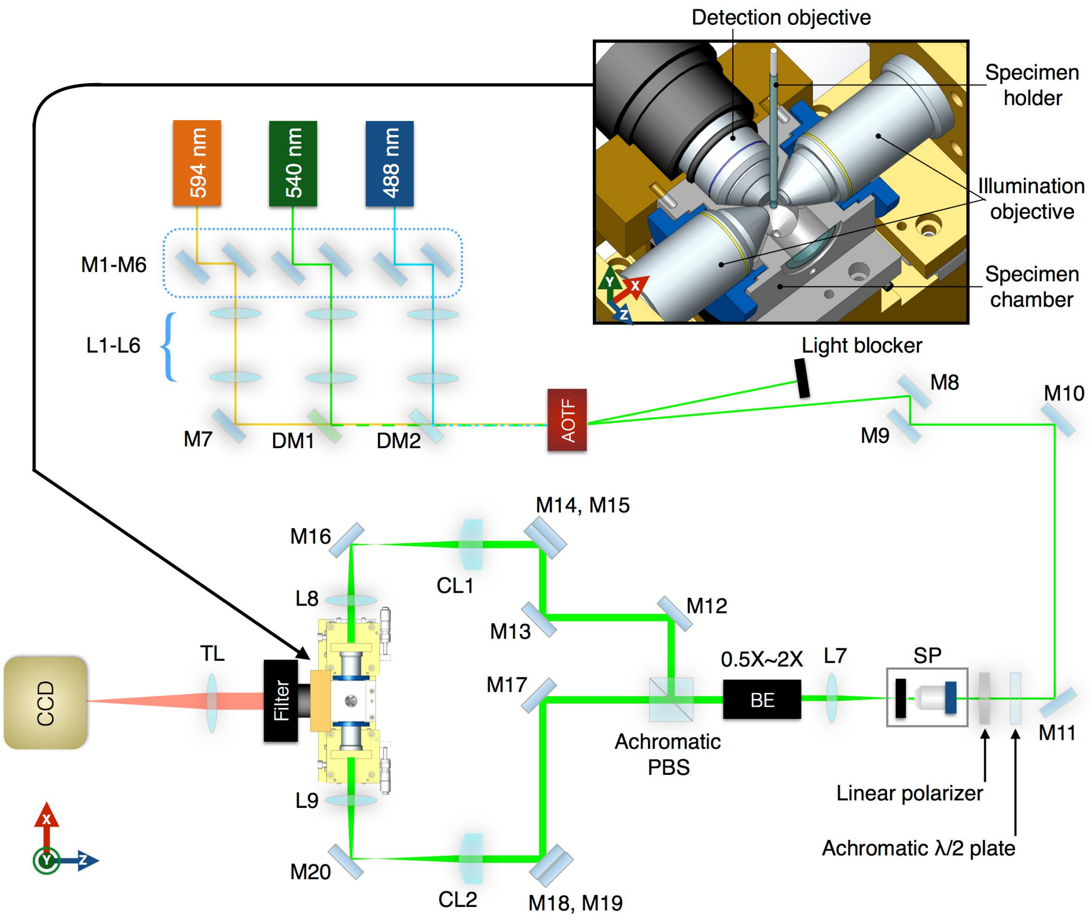

Figure S2. A schematic diagram of the optical setup of a dual-illumination light sheet microscope equipped with three lasers (488, 540, and 594 nm) for florescence excitation. After individually aligning and collimating each laser beam via a set of mirrors (M1-M6, Thorlabs) and a set of lenses (L1-L6, Thorlabs), the laser beams were coupled into an acousto-optic tunable filter (AOTF, AA opto-electronic) to select the appropriate wavelength and intensity. The selected laser beam was directed to a spatial filter (SP, Newport) to refine the beam shape and then collimated again by a lens (L7, Thorlabs). A beam expander (BE, Thorlabs) was employed to adjust the beam size so that the Gaussian light sheet matched the field of view (at the image plane) of the imaging objective lens. The laser beam was split by an achromatic polarized beam splitter (PBS, Thorlabs) into two mutually orthogonal linearly-polarized beams with equal optical power (adjusted by a linear polarizer and a half-wave plate, Thorlabs). Each laser beam was properly relayed by several mirrors (M12, M13 and M17, Thorlabs), raised to an appropriate height by a periscope (M14-15 and M18-19, Thorlabs) and directed into the illumination arm composed of a pair of lenses and an illumination objective. The cylindrical lens (CL1 and CL2, CVI) in the illumination arm formed a 4f-system with an achromatic lens and shaped the light to a thin sheet. The two illumination light sheets propagating in opposite directions were focused by the illumination objectives (Nikon, CFI Plan Fluor 10X, 0.3 NA) and accurately aligned to form the illumination plane where the thinnest laser waists met and coincided in the water-filled specimen chamber. The detection arm was arranged along a direction perpendicular to the illumination plane, such that the later was at the focal plane of the detection objective lens (Nikon, CFI Apo 40XW NIR, 0.8 NA). The fluorescence signals from the illumination plane were collected by the detection objective, filtered (to pass only the desired wavelength) and then imaged on a camera chip (Hamamatsu, ORCA R2) by a tube lens (TL, Thorlabs). For time-lapsed imaging, sample were mounted in the low gelling low melting point agarose and held by a glass capillary suspended in a PBS-filled chamber. The core of the dual-illumination light sheet microscope is enlarged and shown in the upper-right corner to illustrate the configuration of the specimen chamber, sample holder, illumination objectives and the detection objective.

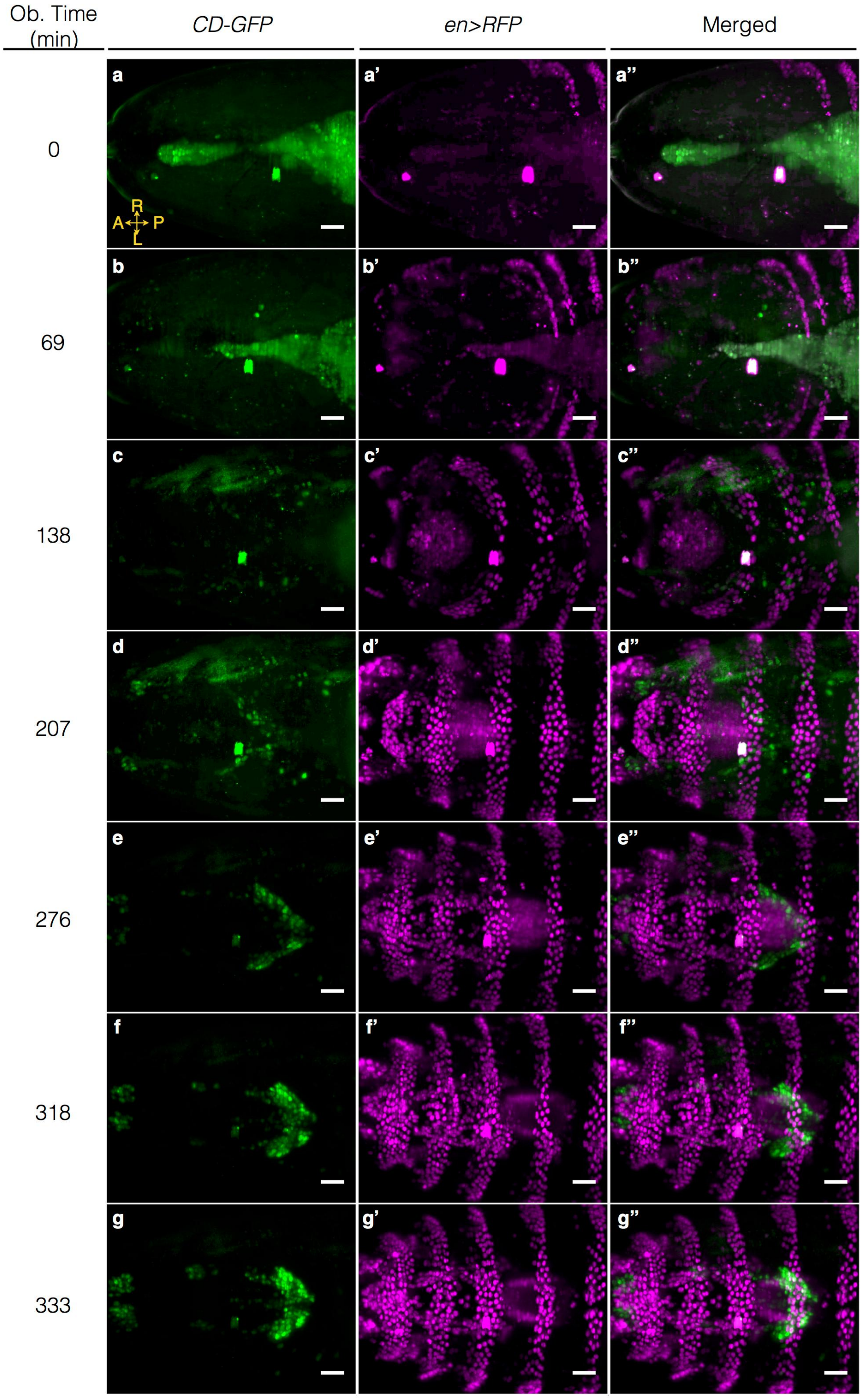

Figure S3. Seven out of 111 time points from a live imaging data set. (**a**) - (**g**) *CD-GFP* (green), (**a'**) - (**g'**) *en>RFP* (magenta), and (**a"**) - (**g"**) merged time-lapsed gene expression micrographs. The observation time points (Ob. Time) of the frames from top to bottom are 0, 69, 138, 207, 276, 318 and 333 min, respectively. The bright V-shape labeled by *CD-GFP* is the EADP. Each image represents the maximum intensity projection from a stack of 84 optical-sectioning 2D images observed from dorsal side. The contrast of each image at different time point had been adjusted individually to accentuate the desired fluorescence signals of interest. Some scattering signals from the vitelline membrane of the embryo and a remnant of its outer chorion, which are irrelevant to our question of interest, were suppressed by treating them as the background noise. Scale bar: 20 μm. The time-lapsed movie can be found as Supplementary Movie 1 online.

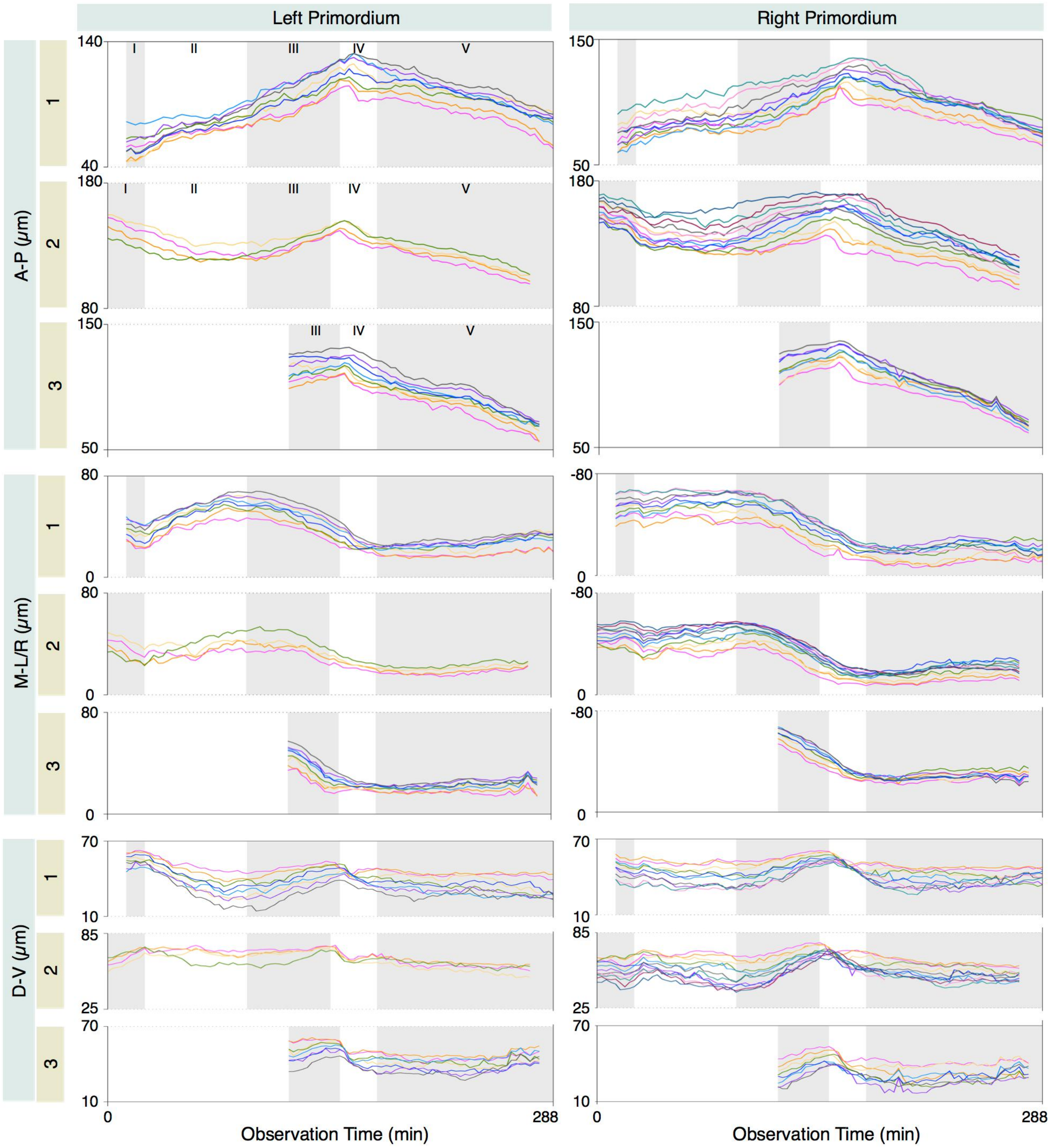

Figure S4. The repeatability of the trajectories of EECs. The trajectories of EECs from three individual embryos (No. 1, 2, and 3) were decomposed along the A-P, M-L/R, D-V axes as described in Fig. 2. To facilitate a comparison of the EECs trajectories in the three individual embryos, each set of trajectories was shifted to align the turning point in the A-P axis in phase IV. The corresponding phases for each embryo are labeled in the upper-left chart. Although the phase periods of different embryos are slightly different, the trajectories show high similarity.

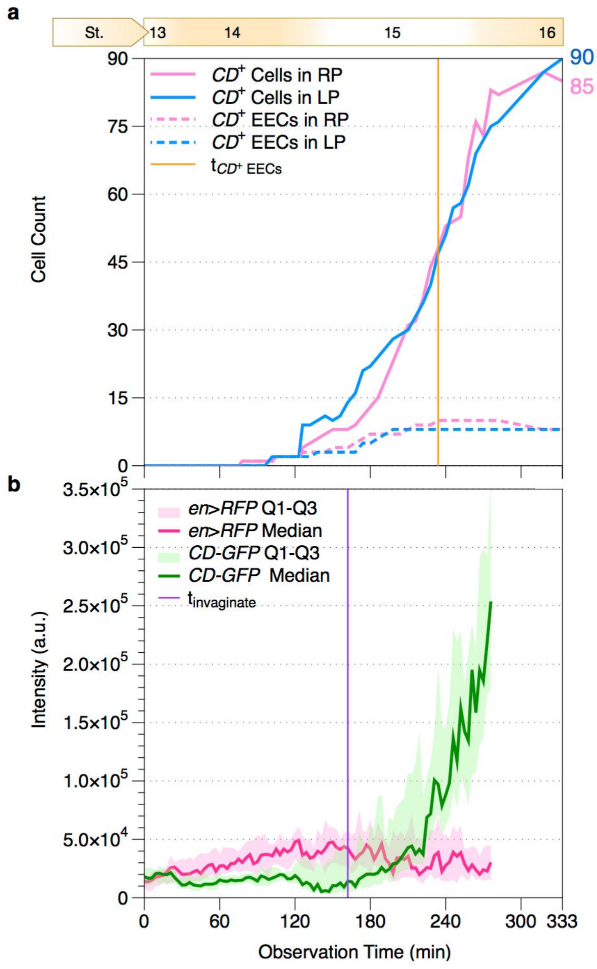

Figure S5. Quantitative analysis of the time-dependent cell numbers and the fluorescence signals of *en*>*RFP* and *CD-GFP* during the course of the EADP formation. (**a**) *CD*$^+$ cell numbers plotted as a function of time. The corresponding embryonic stages (St.) are listed on the top. The pink and the blue lines represent the number of cells in the right and the left primordia (RP, the right primordium; LP, the left primordium), respectively. The solid lines represent the total numbers of *CD-GFP* expressed cells; the dashed lines represent the numbers of the EECs that expressed *CD-GFP* (*CD*$^+$ EECs). The orange vertical line indicates the time when all EECs expressed *CD-GFP* (approximately at the observation time 234 min). At that moment, only nearly half of all EADP cells (95 out of 175 cells in both primordia) have appeared (expressing *CD-GFP*). The cell numbers increased significantly from late stage 14 and reached a plateau in stage 16 (90 cells in the left primordium; 85 cells in the right primordium). (**b**) The time-dependence of the fluorescence intensities of *en*>*RFP* and *CD-GFP* in EECs. The pink and the green lines show the median value of the fluorescence intensities of *en*>*RFP* and *CD-GFP*, respectively. The shading associated with each line shows the data range from the first (Q1) to the third quartiles (Q3). In EECs, the *CD-GFP* intensity continuously increased with time, whereas the *en*>*RFP* intensity slightly decreased. EECs invaginated completely at the observation time 162 min, which is indicated by the purple vertical line. The fluctuation of signals became more serious after invagination because of the interferences from the movement of epidermal segments and the wriggling of the embryo.

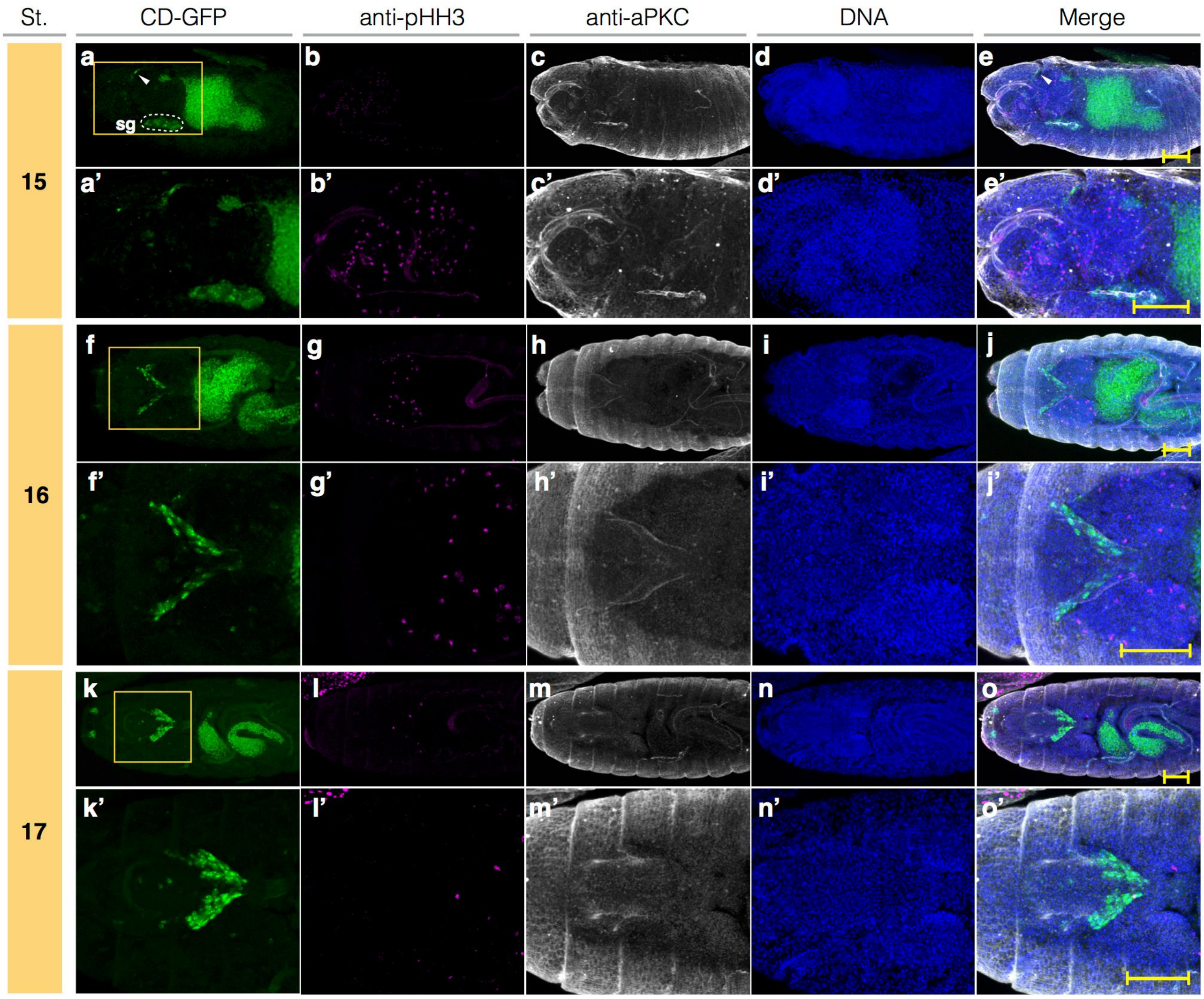

Figure S6. Cell proliferation activity of the EADP in different embryonic stages. (**a**) - (**e**), (**f**) - (**j**) and (**k**) - (**o**) show the mitotic activities of embryos in stage 15, 16 and 17, respectively. *CD-GFP* (green) expressing embryos were co-stained with anti-phospho-histone H3 (anti-pHH3, Cell Signaling 9701, 1:300, magenta), anti-protein kinase C (anti-PKC ζ, Santa Cruz Biotechnology SC-216-G, 1:50, gray) and DAPI (Sigma-Aldrich D9564, 0.5 μg/ml, blue). Each image was the maximum intensity projection (MIP) of a 3D image stack. (**a'**) - (**e'**), (**f'**) - (**j'**) and (**k'**) - (**o'**) show the zoom-in images of the boxed regions in (a), (f), and (k). Merged images show that there were no anti-pHH3 stained cells in the dorsal surface of the procephalic region after stage 15. The mitotic activities were detected only in the central nervous system (CNS); the activities decreased when approaching hatching. The seemingly overlapping signals indicated by arrowheads in (j') and (o') were originated from different planes, other than the image plane of the EADP. Images in the same row are in the same scale. Scale bars are 50 μm. Abbreviation: St., embryonic stage.

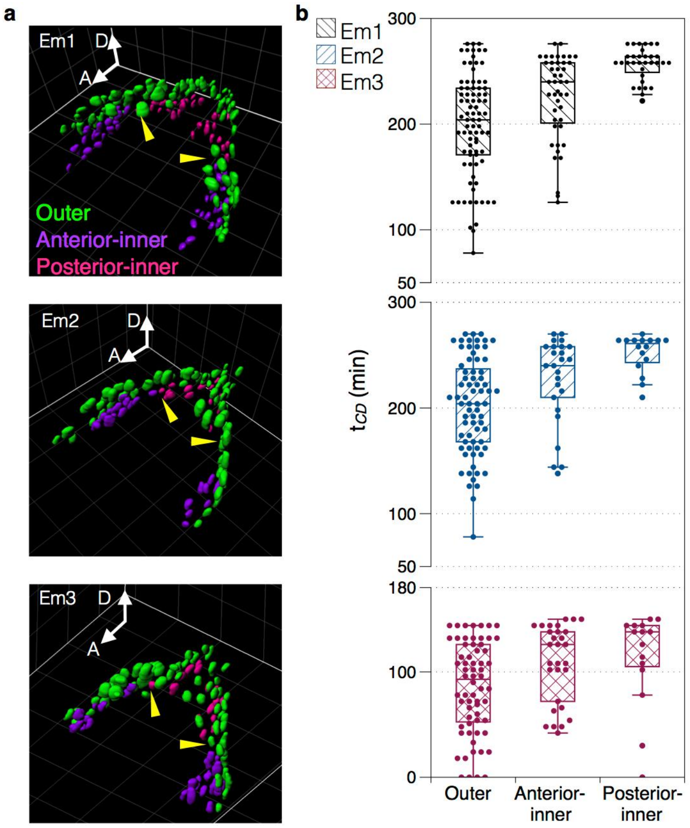

Figure S7. The repeatability of the spatiotemporal relationship analysis of *CD* expression in the EADP. (**a**) The classification of cells in three different regions of the EADP in three individual embryos (Em1, Em2 and Em3) at the latest trackable time points, which are about approximately in stage 16 for all embryos. *CD-GFP* signals from EADP cells were reconstructed by surface rendering and color-coded as was described in Fig. 3. The data from embryo Em2 are the same as those used to obtain the pictures shown in Fig. 3. The two-layer structure can be divided into an outer, an anterior-inner and a posterior-inner region. The anterior and posterior sections in the inner layer of the EADP were demarcated by the waists (indicated by yellow arrowheads). (**b**) Distributions of the onset of *CD-GFP* expression (t_{CD}) of EADP cells. The statistics show a consistent tendency, suggesting that cells in the outer layer, which include most of EECs, expressed *CD-GFP* earlier than those in the inner layer. Grid size in the background is 20 μm.

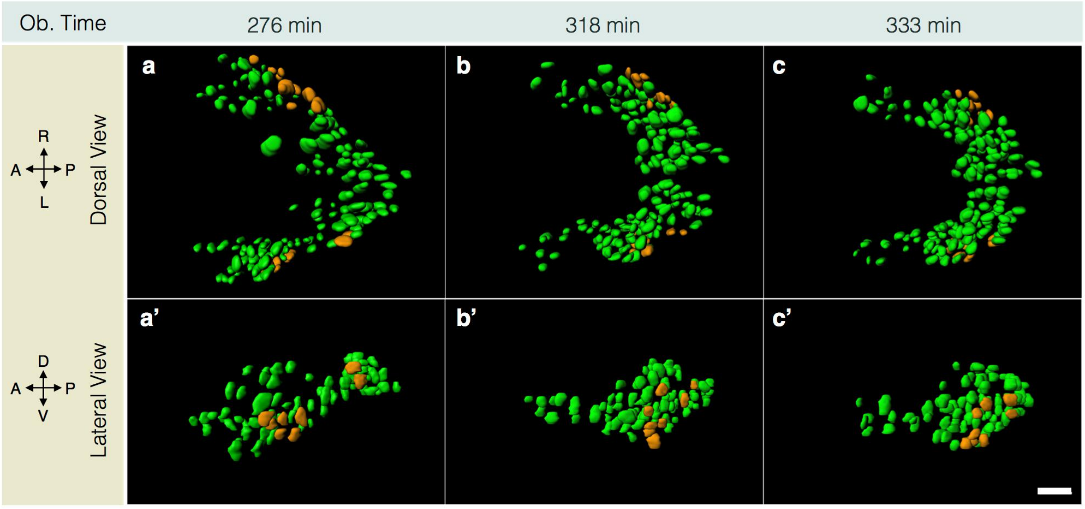

Figure S8. The morphological change of the EADP in late embryonic stage. (**a**, **b**, and **c**) The dorsal view of the surface model of the EADP at the observation time points 276, 318 and 333 min., respectively. (**a', b'** and **c'**) The lateral view of the left primordium at the same observation time as the corresponding image in the same column. The cell number of all CD^+ cells (including EECs) at each time point is 158, 174 and 175, respectively. It shows that the cell number of EADP progenitor cells grew only slightly after the folding, suggesting that the EADP formation was near completion. The shortening and condensing of the EADP indicated that a folding process had taken place to form the sac-like structure of the EADP in late embryonic stage. Ob. Time: the observation time; Scale bar: 20 μm.

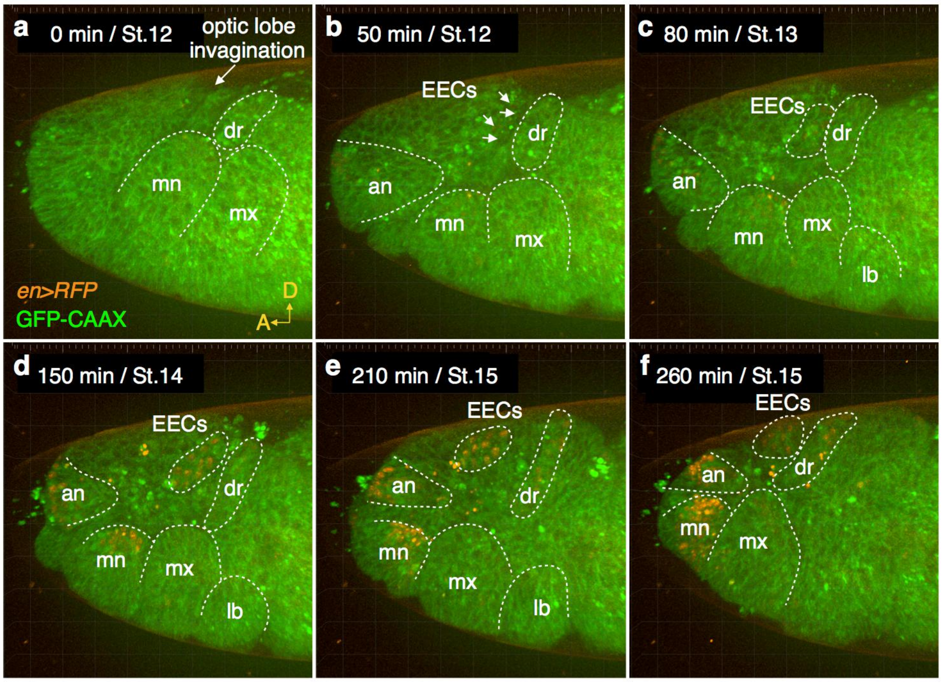

Figure S9. The movements of *en*$^+$ head segments from stage 12 to stage 15. (**a**) – (**f**) Time-lapsed 3D volume rendering of *en>RFP* and *GFP-CAAX* expression patterns in the head region at the observation time 0, 50, 80, 150, 210, and 260 min in lateral view. The corresponding embryonic stages (St.) were listed beside the observation time in the left-upper corner of each frame. *ubi-GFP-CAAX* and *en-Gal4;UAS-H2B-RFP* were crossed. The head morphology was provided by *GFP-CAAX* expression (green). *ubi-GFP-CAAX* expressed ubiquitously and the GFP-CAAX protein marks the cellular membrane and hence shows cell morphology[S1]. EECs and the head segments were specified by *en>RFP* expression (orange) and outlined by dashed lines. (**a**) In the beginning of the observation, the *en>RFP* signal was too weak to be detected. The optic lobe invagination was recognized near the peripheral procephalic lobe and indicated by a white arrow. (**b**) EECs emerged as an independent cell cluster at late stage 12, which was later than the onset of *en* expression (stage 10). The time-delayed detection of *en>RFP* raised the difficulty of tracking the origins of EECs in earlier stage. (**c**) – (**f**) The migration patterns of EECs were consistent with the description in Fig. 2. In contrast, the *en*$^+$ antennal stripe was found to arise independently and stayed laterally and moved posteriorly to invaginate. No *en*$^+$ cells moved from other head segments to join the cell cluster of EECs throughout the observation. The experimental procedure and materials were the same as those reported previously[S2]. Embryos were covered by halocarbon oil and imaged from stage 9 or earlier. The imaging experiment was conducted at 25°C via a spinning disk confocal microscope (Andor, Revolution WD). Each time-lapsed 3D image stack, which contained 73 2D sectioning images, was acquired every 10 min. The detection objective was 40X (Nikon, CFI Plan Fluor 40X Oil, NA 1.3). The time-lapsed 3D images were reconstructed and presented by Imaris (Bitplane Inc.). Embryo anterior is to the left, and the dorsal is up in all frames. Abbreviations: op, optic lobe; dr, dorsal ridge; an, antennal; mn, mandibular; mx, maxillary; lb, labial. Grid size in the background is 20 µm. The time-lapsed movie can be found as Supplementary Movie 6 online.

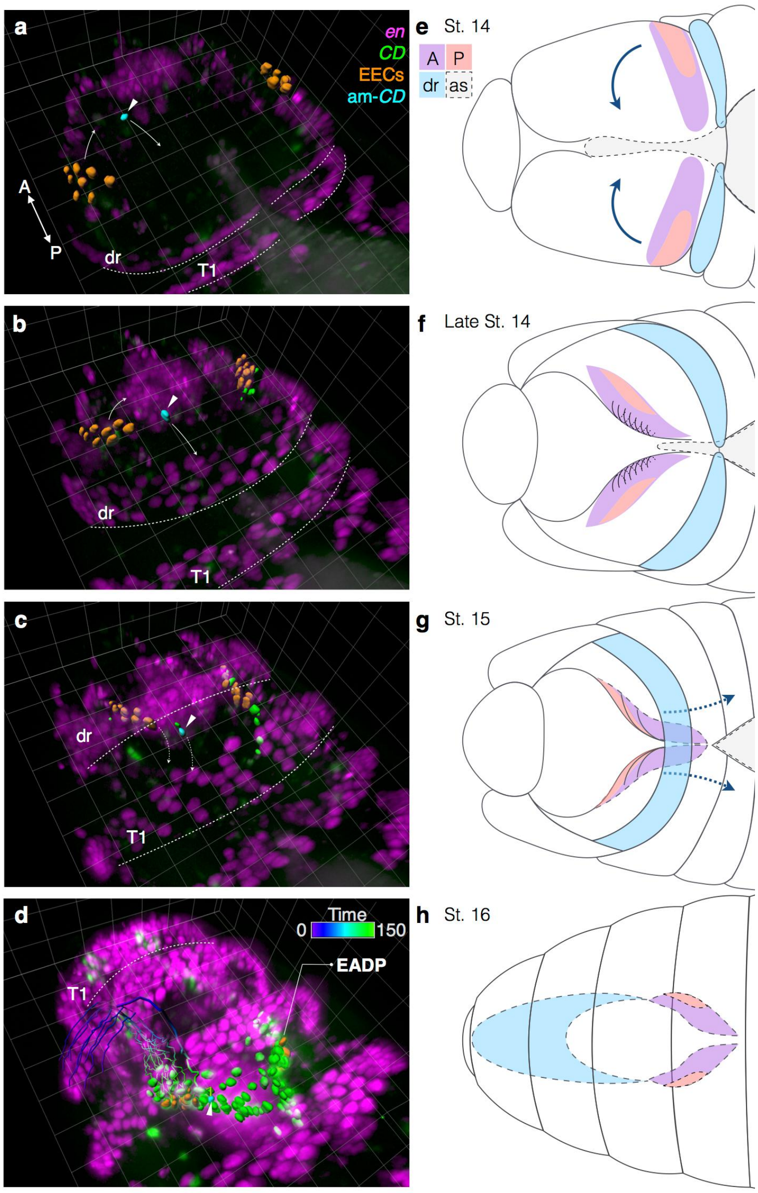

Figure S10. A model suggesting the inversion of anterior and posterior compartments in the EADP. (**a - d**) The cell dynamics to reveal the relative motion of cells in eye-antennal segment. The time intervals relative to (a) were 18, 36, and 150 min in (b), (c), and (d), respectively. The corresponding embryonic stages spanned from late stage 14 to stage 16. Surface renderings of EECs and CD^+ EADP cells are presented in orange and green, respectively. A CD^+ EADP cell located in the anterior medial region of EECs (am-*CD*) in late stage 14 is highlighted in blue and indicated by a white arrow head. The moving directions of EECs and the am-*CD* cell are indicated by white arrow lines. Note that dashed arrows represent the motion in the interior of the embryo. White dashed lines indicate the posterior boundary of the dorsal ridge (dr) and the first thoracic segment (T1). The posterior compartment of the eye-antennal segment is identified by EECs, which had the same migration pattern as described in Fig. 2. The cells in more medial region of the procephalic lobe were pulled posteriorly earlier. The relative motion led to the location change of the am-*CD* cell from anterior to posterior relative to the EECs. The trajectories in (d) show that the am-*CD* cell had a similar migration pattern to that of EECs. The EADP emerged as a V-shape structure in stage 16, in which the dorsal ridge had involuted inside the embryo and hence not shown in (d). (**e - h**) A series of schematic diagrams to illustrate the orientation transposition of the procephalic lobe during the head involution. The arrangement of the anterior (A) and posterior (P) parts of the EADP

rotated by about 90 degrees (indicated by the blue solid arrows) as the EADP cells moved to the dorsal midline from stage 14. The anterior part invaginated into the interior of the embryo and pulled posteriorly ahead of the posterior part in about late stage 14, and the eye-antennal segment was overwhelmed by the dorsal ridge in stage 15. The inference explains the inversion of the A-P compartments in the EADP. Other abbreviations: St., embryonic stage; as, amnioserosa. Grid size in the background is 20 μm.

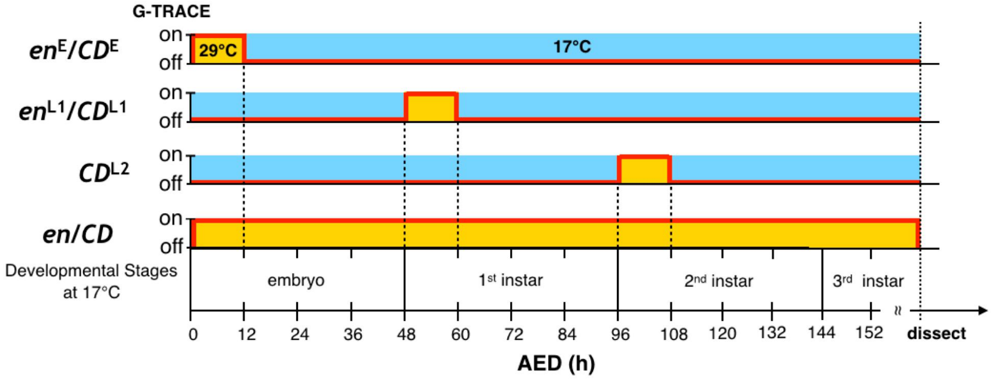

Figure S11. Experimental procedure for stage-specific lineage tracing. The yellow and the blue backgrounds indicate the time periods at 29℃ and 17℃, respectively. To trace the lineage from en^{+} or CD^{+} cells at a certain stage, we used $GAL80^{ts}$ to temporally regulate the *G-TRACE* expression via temperature shift. At 17℃, $GAL80^{ts}$ binds to GAL4 to suppress the GAL4 activity. At 29℃, $GAL80^{ts}$ becomes inactive and unable to inhibit the GAL4 activity. Thus, *en*- or *CD*-driven *G-TRACE* was activated only during the period at 29℃. Since culturing at 17℃ approximately doubled their developmental time, the time after egg deposition (AED) corresponding to each developmental stage was adjusted accordingly. For lineage tracing in embryonic stage (en^{E}/CD^{E}>*G-TRACE*), the eggs were laid and kept at 29℃ for 12 hours, then shifted to 17℃ till dissection. For lineage tracings in the first larval stage (en^{L1}/CD^{L1}>*G-TRACE*) and the second larval stage (CD^{L2}>*G-TRACE*), the eggs were laid and kept at 17℃ for 48 and 96 hours, respectively, and shifted to 29℃ for 12h, then returned to 17℃ till dissection. In the pan-stage lineage experiment (*en*>*G-TRACE* and *CD*>*G-TRACE*), the progeny were kept at 29℃ all the time. Larval EAD in all conditions were dissected in third instar larval stage for fixation and examination.

Supplementary Movie 1: Long-term observation of a *Drosophila* embryo with simultaneous fluorescence expressions of *en* and *CD* enhancer from embryonic stage 13 to 16.

3D volume rendering of a *Drosophila* embryo imaged via dual-illumination light sheet microscopy. The fluorescence signals shown in green and magenta are *CD-GFP* and *en>RFP*, respectively. Disturbance due to the wriggling of the live fly embryo occurred from the observation time 276 min, which was the last time point for cell tracking. Grid size in the background is 20 μm.

Supplementary Movie 2: Cell dynamics during the EADP formation through a 5-phase process.

3D volume rendering of expression patterns of *CD-GFP* and *en>RFP* with 3D surface rendering of fluorescence signals from EADP cell nuclei over the trackable period (the observation time 0 to 273 min). The surfaces shown in green, yellow and orange are cells expressed only *CD-GFP*, EECs and CD^+ EECs, respectively. Grid size in the background is 20 μm.

Supplementary Movie 3: Migration trajectories of CD^+ EADP cells.

The trajectories of EECs and some non-EEC cells expressed *CD-GFP* before the observation time 138 min were reconstructed and color-coded from the observation time 126 min to 276 min. The trajectories of those non-EEC CD^+ cells were highlighted in the form of thicker lines. The similarity of trajectories of the non-EEC CD^+ cells to those of EECs suggested that the CD^+ EADP cells followed the same migration pattern of EECs to invaginate during the EADP formation. Grid size in the background is 20 μm.

Supplementary Movie 4: The spatial-temporal distribution of the CD^+ EADP cells.

CD^+ cells color-coded for their locations in the time-lapsed movie show that the outer layer of EADP cells expressed *CD-GFP* earlier than those in the inner layer. The color-code of each group of EADP cells is the same as was described in Fig. 3. *en*- and *CD*- expression patterns are shown in cyan and green, respectively.

Supplementary Movie 5: The cluster-region of EECs intermixed by CD^+ non-EEC cells.

The trajectories of EECs and two intermixing CD^+ cells (marked in magenta) were reconstructed and color-coded from the observation time 198 min to 276 min. Trajectories of the intermixing CD^+ cells are highlighted in the form of thicker lines. The cluster-region of EECs was trespassed during the EADP formation, suggesting that the boundary property of the *en*-defined posterior compartment may not be strictly retained during the EADP morphogenesis. Grid size in the background is 20 μm.

Supplementary Movie 6: Time-lapsed images of a *CAAX-en* co-expressing embryo in lateral view from stage 12 to stage 15.

A *en>RFP GFP-CAAX* co-expressing embryo was imaged by a spinning disk confocal microscope from stage 12 to stage 15. The intensity of *en* expression pattern increased with time. EECs, at the observation time 50 min, emerged as an independent cell cluster (indicated by white arrows). No en^+ cells moved from other places to join the cell cluster formed by EECs since EECs can be identified. The migration patterns of EECs were the same as described in Fig. 2. In contrast, the en^+ antennal stripe was found to appear independently and stayed laterally and moved posteriorly to invaginate. The migration patterns of en^+ antennal segment and EECs were distinctly different. Abbreviations: op, optic lobe; dr, dorsal ridge; an, antennal; mn, mandibular; mx, maxillary; lb, labial. Grid size in the background is 20 μm.

Reference

S1. Gao, J., Liao, J. & Yang, G.-Y. CAAX-box protein, prenylation process and carcinogenesis. *American Journal of Translational Research* **1**, 312-325 (2009).

S2. Parton, R. M., Vallés, A. M., Dobbie, I. M. & Davis, I. Collection and Mounting of Drosophila Embryos for Imaging. *Cold Spring Harbor Protocols* **2010**, pdb.prot5403, doi: 10.1101/pdb.prot5403 (2010).
